# Bile reflux alters the profile of the gastric mucosa microbiota

**DOI:** 10.3389/fcimb.2022.940687

**Published:** 2022-09-09

**Authors:** Gang Huang, Sui Wang, Juexin Wang, Lin Tian, Yanbo Yu, Xiuli Zuo, Yanqing Li

**Affiliations:** Department of Gastroenterology, Qilu Hospital of Shandong University, Jinan, China

**Keywords:** bile reflux, microbiota, bile acid, 16S rRNA sequencing, bacteria

## Abstract

**Background:**

Bile reflux can cause inflammation, gastric mucosa atrophy, and diseases such as stomach cancer. Alkaline bile flowing back into the stomach affects the intragastric environment and can alter the gastric bacterial community. We sought to identify the characteristics of the stomach mucosal microbiota in patients with bile reflux.

**Methods:**

Gastric mucosal samples were collected from 52 and 40 chronic gastritis patients with and without bile reflux, respectively. The bacterial profile was determined using 16S rRNA gene analysis.

**Results:**

In the absence of H. pylori infection, the richness (based on the Sobs and Chao1 indices; P <0.05) and diversity (based on Shannon indices; P <0.05) of gastric mucosa microbiota were higher in patients with bile reflux patients than in those without. There was a marked difference in the microbiota structure between patients with and without bile reflux (ANOSIM, R=0.058, P=0.011). While the genera, *Comamonas*, *Halomonas*, *Bradymonas*, *Pseudomonas*, *Marinobacter*, *Arthrobacter*, and *Shewanella* were enriched in patients with bile reflux, the genera, *Haemophilus*, *Porphyromonas*, and *Subdoligranulum*, were enriched in those without bile reflux.

**Conclusion:**

Our results demonstrate that bile reflux significantly alters the composition of the gastric microbiota.

## Introduction

Bile reflux, or “duodenogastric reflux,” refers to the retrograde movement of duodenal content (mainly bile) into the stomach resulting from various causes. Mild bile reflux is considered a normal postprandial physiological phenomenon and may occur in healthy individuals. (Koek et al., 2005) However, the regurgitation of duodenal bile contents associated with clinical symptoms and inflammation can result in endoscopic and histologic changes, a condition known as bile reflux gastritis (BRG). Studies indicate that bile is a potential carcinogen that can promote the development of esophageal cancer, gastric cancer (GC), hypopharyngeal cancer, and other serious diseases. (Fein et al., 1998; Li et al., 2018; Sasaki et al., 2019; Li et al., 2020; Vageli et al., 2021).

Until recently, BRG was considered a surgical condition resulting from the removal or alteration of the pylorus, such as Billroth II subtotal gastrectomy. However, BRG is increasingly occurring among individuals who have not had gastrointestinal surgery, a condition known as “primary bile reflux gastritis (PBRG),” indicating that the complex etiology and pathogenesis of bile reflux may be associated with gastrointestinal motility and neuroendocrine involvement.

Sequencing technology has allowed for a more in-depth assessment of the relationship between gastric microbiota and disease pathogenesis. Some studies indicate a potential relationship between bile reflux and certain intragastric bacteria such as H. pylori (HP), but specific gastric flora have not yet been defined. (Ladas et al., 1996; Arslan and Balamtekin, 2021) The movement of alkaline bile back into the stomach may affect gastric acidity and further change the bacterial communities in this region. The current study investigates the alterations and distribution of stomach microbiota in patients with bile reflux.

## Methods

### Study population

The study protocol was approved by the Medical Ethics Committee of Shandong University Qilu Hospital and written informed consent was completed by all included subjects. A total of 92 patients, including 49 males and 43 females who were 48.00 ± 14.26 years of age, were enrolled between December 2020 and July 2021 at Shandong University Qilu Hospital. All patients underwent gastroscopy for a careful inspection of the gastric lumen and, if necessary, a histological examination. Bile reflux, defined as the presence of intragastric bile during endoscopy (Sasaki et al., 2019; Wang et al., 2022), was used to categorize the patients into two groups: (1) chronic gastritis with bile reflux (BR+) patients characterized by the presence of a sufficient amount of intragastric bile in combination with histopathologically identified chronic gastritis and (2) chronic gastritis without bile reflux (BR-) patients characterized by the presence of chronic gastritis without intragastric bile. Patients were excluded if they had additional upper gastrointestinal diseases; All patients in BR+ group had a history of bile reflux confirmed by previous gastroscopy examination before participation in the study; Endoscopy was performed under anesthesia in the early morning in participants who had not taken any food, water, or drugs since the previous night.

### Sample collection and preservation

Two gastric mucosal tissue specimens (N=184) were collected from each of the BR+ and BR- patients. The samples were placed in aseptic sampling tubes and stored at -80°C prior to high throughput sequencing at the laboratory of Majorbio (Shanghai, China).

### DNA extraction and high-throughput sequencing

Total DNA from each sample was extracted using the FastDNA SPIN kit (MP Biomedicals, California, USA). The V3–V4 region of the 16S rRNA gene was amplified by PCR (ABI GeneAmp 9700, ABI, USA) with universal primers (338F: 5′-ACTCCTACGGGAGGCAGCAG-3′ and 806R: 5′-ACTCCTACGGGAGGCAGCAG-3′) using a thermocycler PCR system (GeneAmp 9700, ABI, USA). PCR products were recovered after 2% agarose gel electrophoresis, further purified using gel extraction (AxyPrep DNA GelExtraction Kit, Axygen, California, USA), and quantified by QuantiFluor-ST (Promega, USA) according to the manufacturer’s instructions. Purified amplicons were pooled in equimolar and sequenced on an Illumina MiSeqsystem (Illumina, California, USA).

### Microbial analysis

Raw fastq files obtained from the sequencer were quality-filtered and merged by QIIME (version 1.9.1). Data analysis was performed using the free online platform, Majorbio (www.i-sanger.com). Operational taxonomic units (OTUs) were assigned from the reads at 97% identity using UPARSE (version 7.0, www.drive5.com/uparse/). OTUs were picked using the RDP Classifier algorithm (rdp.cme.msu.edu/) against the SILVA rRNA database (www.arb-silva.de) and pre-clustered at 70% identify. The OTU abundance was normalized using a standard sequence number based on the least sequences of the samples. Within-sample diversity (alpha diversity) was obtained to assess species diversity in locally homogeneous environments. Principal coordinate analysis (PCoA) was used for between-sample diversity (beta diversity) to evaluate species diversity in different environments. Linear discriminant analysis (LDA) effect size (LEfSe) was used to identify significant differences in relative abundance between the groups. The data presented were expressed as the means ± SD. The effects of gender, HP infection, and mucosal atrophy were evaluated using the Chi-square test. The Kolmogorov-Smirnov test was performed to contrast the normality of the distribution between different groups. The student’s t-test or Wilcoxon rank-sum test was used to compare continuous variables. Differences were considered significant when P <0.05. SPSS 25.0 software was used for the statistical analysis.

## Results

### Participant characteristics

Fifty-two participants were BR+, of whom 30 were male, 23 were HP-positive, and 23 had gastric atrophy, while 40 participants were BR-, of whom 19 were male, 15 were HP-positive, and 20 had gastric atrophy study (**Table 1**). No significant differences in gender (male: 57.7% vs. 47.5%, P=0.740) or age (48.35 ± 1.93 vs. 47.55 ± 2.35, P=0.792) were found between the BR+ and BR- groups, respectively.

**Table 1:** Characteristics of the study participants.

	BR+	BR-	*P* value
	(n = 52)	(n = 40)
Age, y (mean±SD)	48.35 ± 1.93	47.55 ± 2.35	0.792
Sex, n (%)			0.740
Male	30 (57.7)	19 (47.5)	
Female	22 (42.3)	21 (52.5)	
HP infection, n (%)			0.516
Positive	23 (44.2)	15 (37.5)	
Negative	29 (55.8)	25 (62.5)	
Atrophy, n (%)			0.582
Yes	23 (44.2)	20 (50.0)	
No	29 (55.8)	20 (50.0)	

HP infection determined by 16 s RNA sequencing.

To further study the relationship between bile reflux and HP infection, the patients were further divided into the following four groups (**Supplementary Table 1**): those with bile reflux and HP infection (BR+ HP+), those with bile reflux without HP infection (BR+ HP-), those with HP infection without bile reflux (BR- HP+), and those with neither (control group; BR- HP-). The relationship between bile reflux and gastric atrophy was also further explored. To avoid potential confounding by HP infection, only patients who were HP-negative according to 16S-RNA sequencing were included in this assessment and divided into the following four groups (**Supplementary Table 2**): those with bile reflux with gastric atrophy (BR+ AG), those with bile reflux without gastric atrophy (BR+ NAG), those with gastric atrophy without bile reflux (BR- AG) and those with neither (control group; BR- NAG). No significant differences were observed associated with bile reflux between these subgroups.

### Patients with bile reflux have higher gastric bacterial diversity

**Figure 1:**
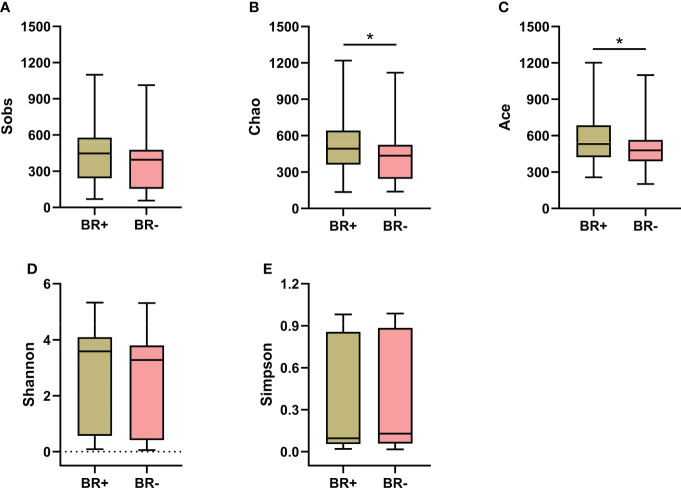
Alpha diversity of the gastric microbiota in the BR+ and BR- groups. The Wilcoxon rank-sum test was used to compare the Sobs **(A)**, Chao1 **(B)**, Ace **(C)**, Shannon **(D)**, and Simpson **(E)** indices between the two groups. *, P < 0.05.

To characterize the gastric microbiota of patients with bile reflux, microbial richness and diversity were assessed based on the hypervariable V3–V4 regions of the 16S ribosomal RNA gene. OTU clustering of the sequence was determined at 97% similarity, and 4,820 OTUs were obtained, with an average of 52 OTUs per sample. The alpha diversity of the microbiota was estimated and the mean values were compared between groups (**Figure 1**). The microbiota of the BR+ group had higher Chao1- and Ace- estimated microbial richness than the microbiota of the BR- group (P <0.05 for both). The Shannon and Simpson indices indicated that the BR+ group had slightly higher diversity than the BR- group but this was not statistically significant.

**Figure 2:**
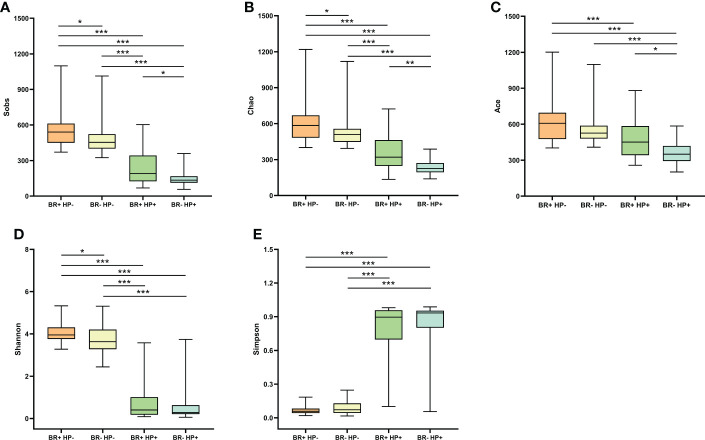
Alpha diversity of the gastric microbiota in the BR+ HP-, BR- HP-, BR+ HP+, and BR- HP+ groups. The Wilcoxon rank-sum test was used to compare the Sobs **(A)**, Chao1 **(B)**, Ace **(C)**, Shannon **(D)**, and Simpson **(E)** indices between the groups. *, P < 0.05; **, P < 001; ***, P < 0.001.

The microbiota of the BR+ HP+ group had significantly reduced microbial richness (based on the Chao1, Sobs, and Ace indices; P <0.001) and diversity (based on the Shannon and Simpson indices; P <0.001) than the microbiota of the BR+ HP- group (**Figure 2**). Similarly, the microbiota of the BR- HP+ group also had significantly reduced microbial richness (based on the Chao1, Sobs, and Ace indices; P <0.001) and diversity (based on the Shannon and Simpson indices; P <0.001) than the BR- HP- group. In addition, the microbiota of the BR+ HP+ group had higher Sobs-, Chao1-, and Ace- estimated microbial richness than the BR- HP+ group (P <0.05, P <0.01, and P <0.05, respectively). The microbiota of the BR+ HP- group had higher Chao1- and Sobs- estimated microbial richness than the microbiota of the BR- HP- group (P <0.05 for both). The BR+ HP- group also had higher microbial diversity than the BR- HP- group (based on the Shannon indices; P <0.05).

**Figure 3:**
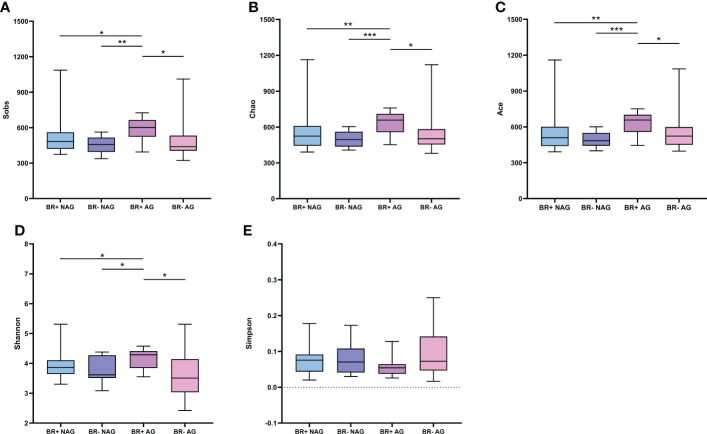
Alpha diversity of the gastric microbiota in the BR+ NAG, BR- NAG, BR+ AG, and BR- AG groups. The Wilcoxon rank-sum test was used to compare the Sobs **(A)**, Chao1 **(B)**, Ace **(C)**, Shannon **(D)**, and Simpson **(E)** indices between the groups. *, P < 0.05; **, P < 001; ***, P < 0.001.

The microbiota of the BR+ AG group had higher Sobs-, Chao1- and Ace- estimated microbial richness than the BR- AG group (**Figure 3**; P<0.05 for all). The BR+ AG group also had higher microbial diversity than the BR- AG group (based on the Shannon indices; P <0.05). While the BR+ NAG microbiome had a slightly higher richness and diversity than the BR- NAG microbiome based on the Sobs, Chao1, Ace, Shannon, and Simpson indices, this was not statistically significant.

### Bile reflux alters the microbiota structure

**Figure 4:**
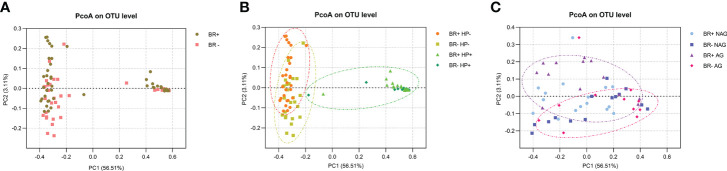
PcoA analysis of the gastric microbiota. **(A)** Comparison of the BR+ and BR- groups, **(B)** Comparison of the BR+ HP-, BR- HP-, BR+ HP+ and BR- HP+ groups; orange circle, a gastric sample from the BR+ HP- group; yellow circle, a gastric sample from the BR- HP- group; green circle, a gastric sample from a patient with HP infection. **(C)** Comparison of the BR+ NAG, BR- NAG, BR+ AG, and BR- AG groups; purple circle, a gastric sample from the BR+ AG group; red circle, a gastric sample from the BR- AG group.

Principal coordinates analysis (PCoA) was used to estimate beta diversity and evaluate the similarity between the bacterial community structures and the overall microbial composition (**Figure 4**). No significant differences were found in the microbiota structures of the BR+ and BR- groups (ANOSIM, R=0.019, P=0.157). While the gastric mucosal microbiota structure differed significantly between the BR+ HP- and BR- HP- groups (ANOSIM, R=0.058, P=0.011), there was no significant difference in structure between the BR+ HP+ and BR- HP+ groups (ANOSIM, R=0.028, P=0.373). In addition, while the gastric mucosal microbiota structure differed significantly between the BR+ AG and BR- AG groups (ANOSIM, R=0.1018, P=0.026), there was no significant difference between the BR+ NAG and BR- NAG groups (ANOSIM, R=0.0641, P=0.059).

### Bile reflux alters the composition of the gastric mucosa microbiota

**Figure 5:**
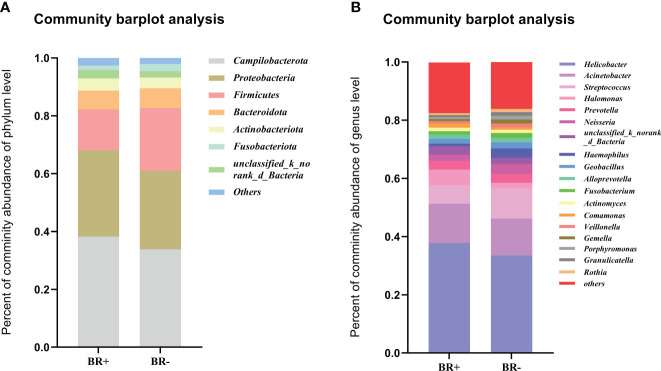
The abundance of relative taxa in the gastric microbiota. Comparison of the abundance of relative taxa between the BR+ and BR- groups at the **(A)** phylum and **(B)** genus level.

In this study, the most abundant phyla of the gastric microbiota were *Campilobacterota*, *Proteobacteria*, *Firmicutes*, *Bacteroidota*, and *Actinobacteriota* (**Figure 5A**). Seventeen genera comprised up to 80% of the total gastric microbiota: *Helicobacter*, *Acinetobacter*, *Streptococcus*, *Halomonas*, *Prevotella*, *Neisseria*, *Haemophilus*, *Geobacillus*, *Alloprevotella*, *Fusobacterium*, *Actinomyces*, *Comamonas*, *Veillonella*, *Gemella*, *Porphyromonas*, *Granulicatella*, *Rothia* (**Figure 5B**).

**Figure 6:**
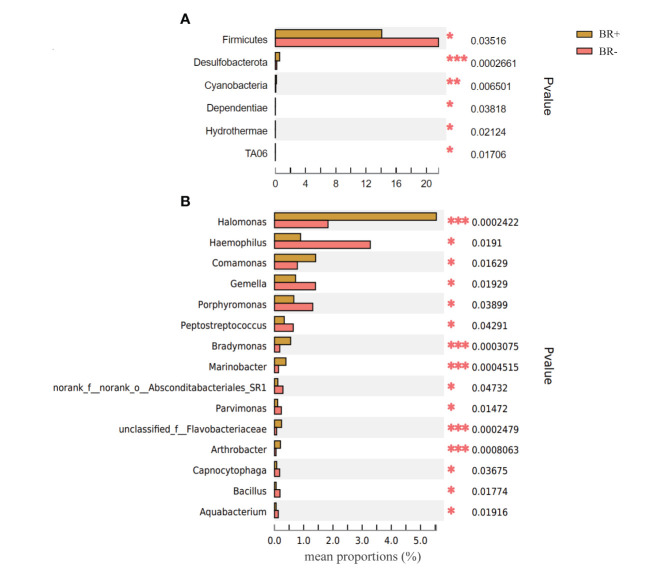
Comparison of the abundance of relative taxa between the BR+ and BR- groups at the **(A)** phylum and **(B)** genus levels *, P <0.05; **, P <001; ***, P <0.001.

**Figure 7:**
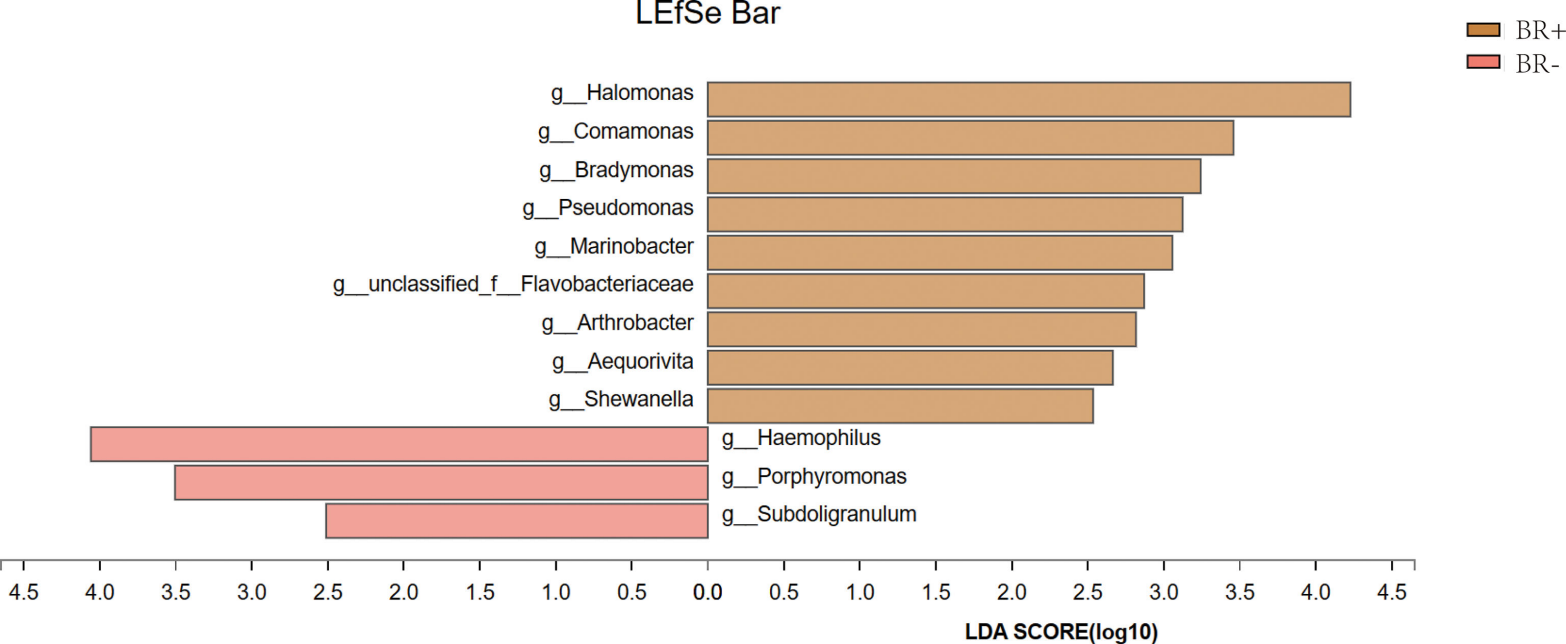
The most differentially abundant taxa between the BR+ and BR- groups using LEfSe analysis.

The gastric microbiota of the BR+ and BR- groups were compared at the phylum and genus levels (**Figure 6**), and LDA effect size was used to identify specific taxa that most likely contributed to the differences between the BR+ and BR- groups (**Figure 7**). The genera *Halomonas*, *Comamonas*, *Bradymonas*, *Pseudomonas*, *Marinobacter*, *Arthrobacter*, and *Shewanella* were enriched in the BR+ group while the genera *Haemophilus*, *Porphyromonas*, and *Subdoligranulum* were enriched in the BR- group. In addition, LEfSe analysis showed that differences in these genera also existed between patients with and without bile reflux, whether or not this condition was complicated by HP infection (**Supplementary Figures 1**, **2**) or gastric mucosal atrophy (**Supplementary Figures 3**, **4**).

## Discussion

Gastric juice secretion results in high stomach acidity, and this unique ecological environment fosters the development of a specific microbial community. Recent studies using high-throughput sequencing of gastric flora have confirmed the presence of complex bacterial flora in the stomach. However, there is little data on how the gastric microbiome changes during bile reflux. In the current study, the bacterial flora of patients with endoscopic bile reflux was more diverse, distinctly structured, and had different taxa than the flora of patients with no endoscopic bile reflux. These findings indicate that patients with endoscopic bile reflux have a distinct gastric microbiome than those without bile accumulates in the stomach, and these differences may impact the occurrence and development of PBRG.

The genera, *Comamonas*, *Halomonas*, *Bradymonas*, *Pseudomonas*, *Marinobacter*, *Arthrobacter*, and *Shewanella* were enriched in patients with bile reflux. *Comamonas* is a cellulolytic microbe associated with inflammation (Kanokratana et al., 2018), and a recent study suggests that a stepwise increase in the abundance of *Comamonas* is associated with the occurrence and development of gastric cancer. (Dai et al., 2021) This genus affects sugar metabolism and is involved in amino acid, carbohydrate, nucleoside, and nucleotide synthesis. (Dai et al., 2021) *Comamonas* impacts the metabolism of cancer patients and may reduce their response to chemotherapeutics such as 5-fluoro-2’-deoxyuridine. (García-González et al., 2017)

*Halomonas* and *Shewanella* thrive in alkaline pH and high salt concentration environments. *Halomonas* has been associated with the pathogenesis of human disease by causing bacteremia and hemodialysis, which requires salt to prepare dialysate with appropriate osmotic pressure. (Kim et al., 2013) *Shewanellae* is also being increasingly linked to bacteremia, soft tissue infections, and otitis media. (Janda and Abbott, 2014) A recent study found that *Halomonas* and *Shewanella* were significantly higher in the peritumoral regions of GC patients than in healthy people, indicating a potential role for these two genera in the occurrence of GC (Ling et al., 2019) The abundance of *Halomonas* is also significantly higher in the gut flora of patients with bronchial asthma (Tikunov et al., 2021) and is linked to HIV-associated microbiome alterations (Xu et al., 2021).

*Pseudomonas* is an opportunistic pathogen associated with inflammation. The abundance of this genus has been shown to increase in inflamed and eroded mucous membranes of the esophagus, stomach, and duodenum. (Chervinets et al., 2020) *Pseudomonas aeruginosa* is also shown to promote gastric tumorigenesis in colonized rats. (Morishita and Shimizu, 1983) Increases in the abundance of this and other bacteria in patients with bile reflux may reflect a potential relationship between the bile reflux-specific microbiome and the occurrence and development of GC. Further studies are required to explore this association.

Most gastric cancers develop from gastritis, atrophy, intestinal metaplasia (IM), and dysplasia. Bile reflux is positively correlated with the severity of glandular atrophy and chronic inflammation and is considered a potential carcinogen. (Sobala et al., 1993; Mercan et al., 2016) Gastric IM is the replacement of normal gastric epithelium with intestinal-like cells and is thought to be driven by chronic environmental stimuli such as bile acid reflux-associated inflammation. (Jiang et al., 2017) While studies indicate that the incidence of bile reflux is positively associated with the occurrence of IM, (Jiang et al., 2017) the mechanism for this association is still being explored. Intragastric bile increases IM biomarkers by activating bile acid receptors, including the farnesoid X receptor and G-protein-coupled receptor 5. (He et al., 2022) Some studies indicate that bile acid-associated IM induces miR-92a-1–5p expression by targeting FOXD1 and activating nuclear factor kappa B (NF-κB) signaling. (Li et al., 2019; Yue et al., 2021) Exosomes, membrane vesicles that are secreted by macrophages, act on gastric epithelial cells and are also involved in bile acid-induced IM (He et al., 2022).

A recent study found that bile reflux had no significant effect on the gastric bacterial microbiota, which seemed inconsistent with our results. (Yang et al., 2022) Further subgroup analysis in our study may reveal the reason for this “inconsistency”. While bile reflux had little effect on the diversity and structure of gastric mucosa microflora in patients with non-atrophy gastritis, it greatly increased microbial diversity and changed the structure of gastric mucosa microflora in those with atrophic gastritis. Atrophy of the gastric mucosa often results in the lack of parietal cells and reduced gastric acid secretion. Shrinking gastric glands and intragastric bile combine to induce a dramatic increase in stomach pH levels, accentuating differences in the bacterial flora composition. In addition, the change of acidity in stomach affected the physicochemical properties of bile acids. Studies have shown that most bile acids precipitate at a low pH and cause more damage to gastric mucosa at a higher pH. (McCabe and Dilly, 2018) The effects of bile reflux on gastric damage and bacterial flora may be more significant at lower acidity.

Studies suggest that there is no significant difference in the composition of the gastric flora between patients with atrophic and non-atrophic gastritis, (Zhang et al., 2021) which is consistent with our results. However, a significant difference was found in the diversity of the microflora in patients with atrophic and non-atrophic gastritis when bile reflux was involved (**Figure 3D**). This result suggests that mucosal atrophy caused by bile reflux has specific microbiological features. LEfSe analysis indicates that patients with atrophic gastritis have a higher abundance of *Acholeplasma* in the gastric mucosa, while those with non-atrophic gastritis have a higher abundance of *Neisseria*, *Actinobacillus*, and *Porphyromonas* (**Supplementary Figure 5**).

Bile acids inhibit the growth, attachment, and colonization of HP *in vitro*. (Mathai et al., 1991) Additional studies suggest that bile inhibits the growth of HP in a dose-dependent manner and increasing acidity (pH ≤5) significantly weakens this inhibition. (Graham and Osato, 2000) The impact of bile reflux on HP infection in the real world requires further research. While some clinical studies (O'Connor et al., 1986) suggest that bile reflux reduces HP infection, the bile reflux patients included in these studies often included those undergoing gastrectomy. Since subtotal gastrectomy can significantly reduce HP colonization (Offerhaus et al., 1989), our study was limited to patients with PBRG. Results showed that chronic gastritis patients with and without bile reflux had similar HP infection rates (44.2% vs. 37.5%, respectively; P=0.516). Studies have also found that HP infection aggravates bile reflux and that there is a decrease in bile reflux after HP eradication. (Huang et al., 2018) It has been reported that HP may aid the development of PBRG by reducing the number of somatostatin producing cells and increasing gastrin release, impairing antral and duodenal motility (Calam and Tracy, 1980).

In our study, HP appears to be the most influential member of the gastric flora, having the highest relative abundance when it exists, and associated with higher microfloral diversity in the stomach when it is absent (**Supplementary Figure 6**). These results are consistent with those from previous studies. (Das et al., 2017; Zheng et al., 2021) *Proteobacteria*, *Firmicutes*, *Actinobacteria*, *Bacteroidetes*, and *Fusobacteria* are the dominant gastric phyla regardless of HP infection. For these reasons, focusing on HP-negative gastritis may reflect the interaction between bile reflux and gastric flora. Our findings showed that there was no significant difference in the beta diversity of the BR+ and BR- groups, but obvious differences between the floral structure of the BR+ HP- and BR- HP- groups. This suggests that there is a correlation between bile reflux and the microbial composition of the stomach. The relationship between HP and bile reflux deserves further investigation. Some studies had suggested that the bacterial flora in the stomach can recover to a considerable extent after successfully eradicating HP. (He et al., 2019; Mao et al., 2021) Comparison of the microbiota before and after HP eradication in bile reflux patients with HP infection may help to reveal the potential relationship between HP, bile reflux and gastric microbiota.

Diet and environmental factors can have an important influence on the dominant genera and composition of the stomach microflora. (Ianiro et al., 2015; Das et al., 2017; Yan et al., 2020) We found that both BR+ and BR- patients had a high abundance of halophilic bacteria such as *Halomonas*, *Bradymonas*, *Marinobacter*, and *Shewanella*, which may be linked to a preference for high-salt or pickled foods by coastal residents of China. (Ren et al., 2018) In addition, the higher abundance of halophilic bacteria in patients in the BR+ group than those in the BR- group suggests that eating salty or pickled foods may be a risk factor for bile reflux. Prior studies indicate a potential relationship between the development of gastroesophageal reflux and the consumption of salt or salted foods (Dağlı and Kalkan, 2017).

The etiology and pathogenesis of PBRG remain unclear. Results from the current study indicate that gastric flora may be an important factor in the occurrence and development of PBRG. (Ladas et al., 1996; Arslan and Balamtekin, 2021; Wang et al., 2022) The current medical treatment of PBRG such as proton pump inhibitors, ursodeoxycholic acid, hydrotalcite and prokinetic drugs have limited effects on endoscopic appearance and histological changes, while potential influence on gastrointestinal microbiota. (Tsuda et al., 2015; McCabe and Dilly, 2018; Pearson et al., 2019) Findings suggest that probiotic treatment significantly alters the diversity, community structure, and composition of the gastric microbiota. (Yuan et al., 2021) However, further studies are needed to assess the impact of treating PBRG by regulating the gastric flora using microbiological modulating therapies such as probiotics.

There are some limitations to this study on the relationship between bile reflux and gastric mucosa flora. A control group without endoscopic or histopathological changes and bile reflux was not enrolled, considering that the age of this population was significantly younger than that of the BR+ group, which might have an impact on the gastric microbiota. (Brawner et al., 2017) In addition, this is a cross-sectional study and so cannot define a causal relationship between bile reflux and gastric flora.

## Conclusion

Changes in the gastric microbiota were found in patients with bile reflux. The detection of elevated levels of *Comamonas*, *Halomonas*, *Shewanella*, and other genera may provide a basis for establishing a set of microbiota-based biomarkers for the diagnosis of PBRG in this population. Gastric microbiota changes associated with bile reflux might involve the pathogenesis of PBRG and serve as a potential therapeutic target for this disease.

## Data availability statement

The data presented in the study are deposited in the NCBI Sequence Read Archive repository, accession numbers PRJNA868297.

## Ethics statement

The studies involving human participants were reviewed and approved by Medical Ethics Committee of Qilu Hospital of Shandong University. The patients/participants provided their written informed consent to participate in this study.

## Author contributions

XZ and YY designed the study. GH and SW collected all the tissue samples. JW and YL performed the data analysis. GH and LT wrote the manuscript. All authors have read and critically revised the manuscript. All authors contributed to the article and approved the submitted version.

## Funding

This work was supported by the National Natural Science Foundation of China (grant number NSFC 81670486, 82070540 to YY) and the Fundamental Research Funds of Shandong University (grant number 2017JC036 to YY).

## Conflict of interest

The authors declare that the research was conducted in the absence of any commercial or financial relationships that could be construed as a potential conflict of interest.

## Publisher’s note

All claims expressed in this article are solely those of the authors and do not necessarily represent those of their affiliated organizations, or those of the publisher, the editors and the reviewers. Any product that may be evaluated in this article, or claim that may be made by its manufacturer, is not guaranteed or endorsed by the publisher.

## Supplementary material

Supplementary Figure 1 The most differentially abundant taxa between the BR+ HP- and BR- HP- groups using LEfSe analysis.

Supplementary Figure 2 The most differentially abundant taxa between the BR+ HP+ and BR- HP+ groups using LEfSe analysis.

Supplementary Figure 3 The most differentially abundant taxa between the BR+ NAG and BR- NAG groups using LEfSe analysis.

Supplementary Figure 4 The most differentially abundant taxa between the BR+ AG and BR- AG groups using LEfSe analysis.

Supplementary Figure 5 The most differentially abundant taxa between the BR+ NAG and BR+ AG groups using LEfSe analysis.

Supplementary Figure 6 The abundance of relative taxa in the gastric microbiota. Comparison of the abundance of relative taxa in the BR+ HP-, BR- HP-, BR+ HP+ and BR- HP+ groups at the **(A)** phylum and **(B)** genus level.
