# Supplementary figures and images for: Bile reflux alters the profile of the gastric mucosa microbiota
Source: Front Cell Infect Microbiol. 2022 Sep 9;12:940687. doi: 10.3389/fcimb.2022.940687 (PMC9500345; doi:10.3389/fcimb.2022.940687)

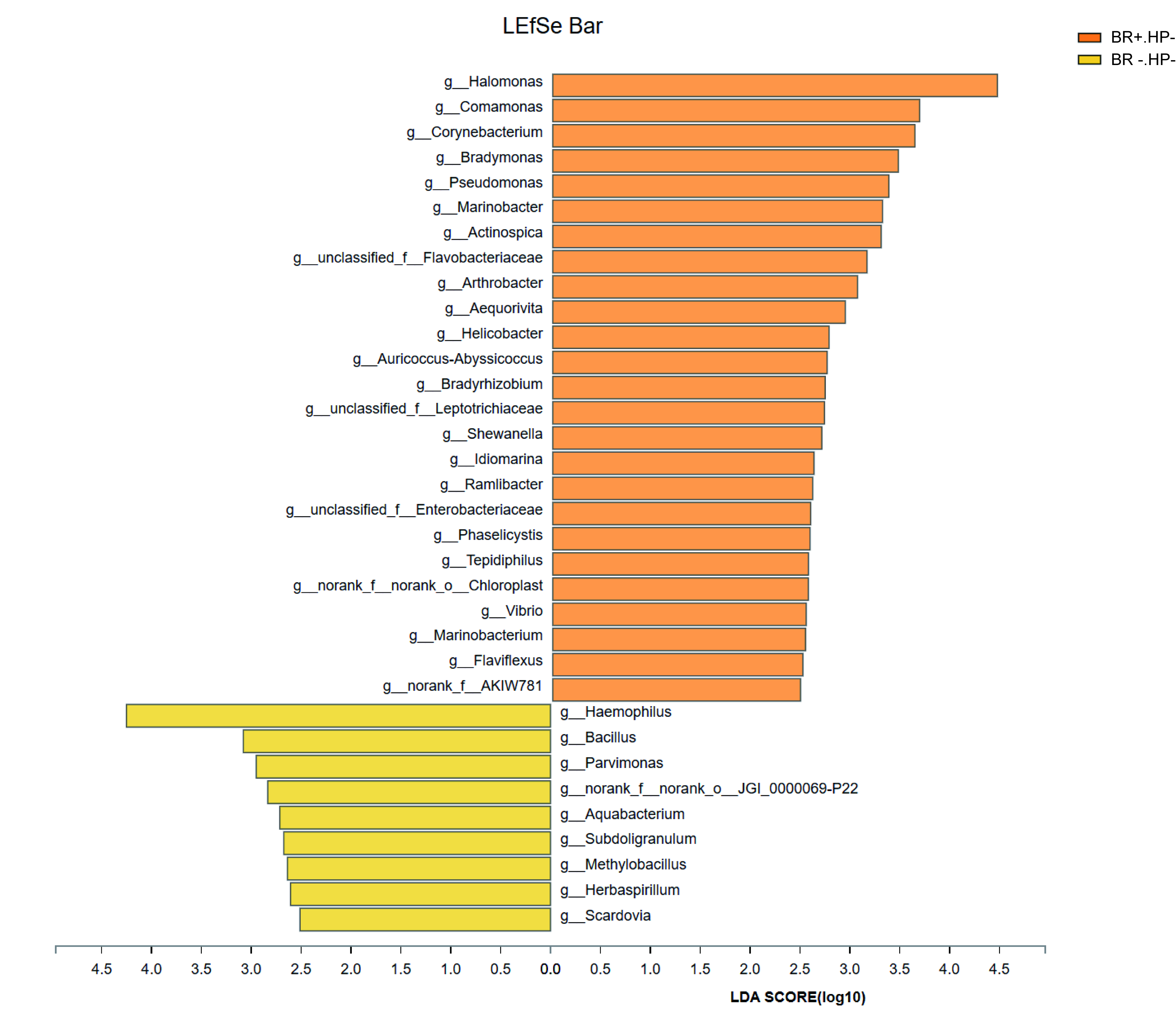

Supplement: Supplementary Figure 1 — The most differentially abundant taxa between the BR+ HP- and BR- HP- groups using LEfSe analysis. [file Image_1.tif]

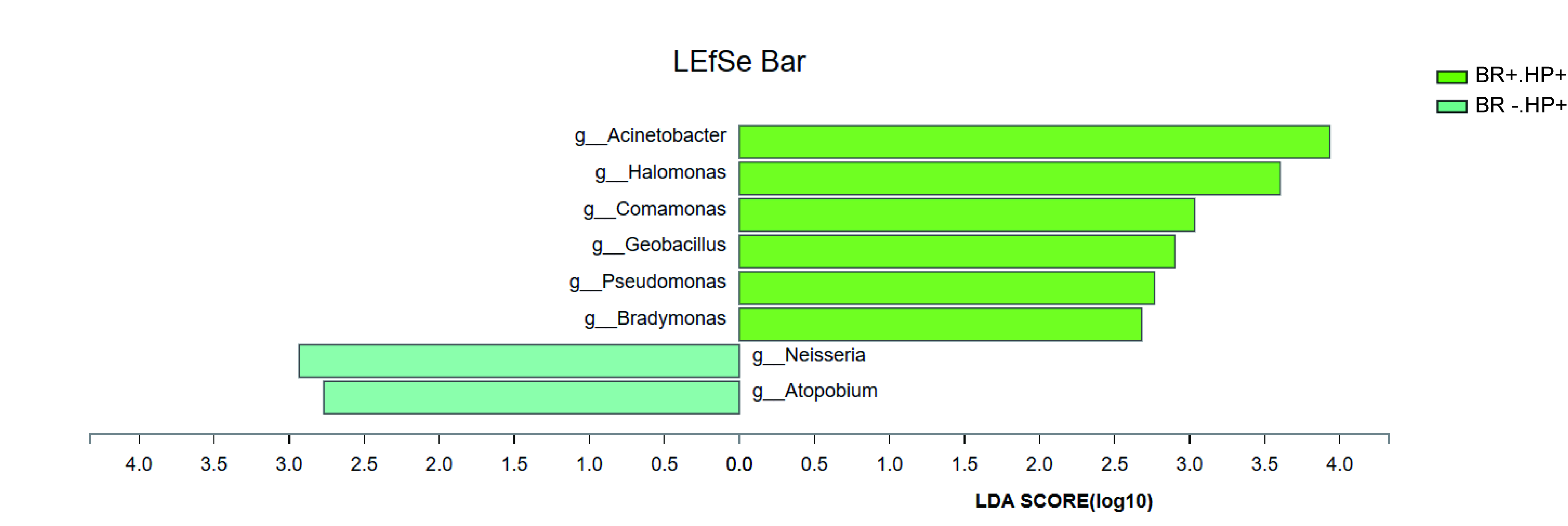

Supplement: Supplementary Figure 2 — The most differentially abundant taxa between the BR+ HP+ and BR- HP+ groups using LEfSe analysis. [file Image_2.tif]

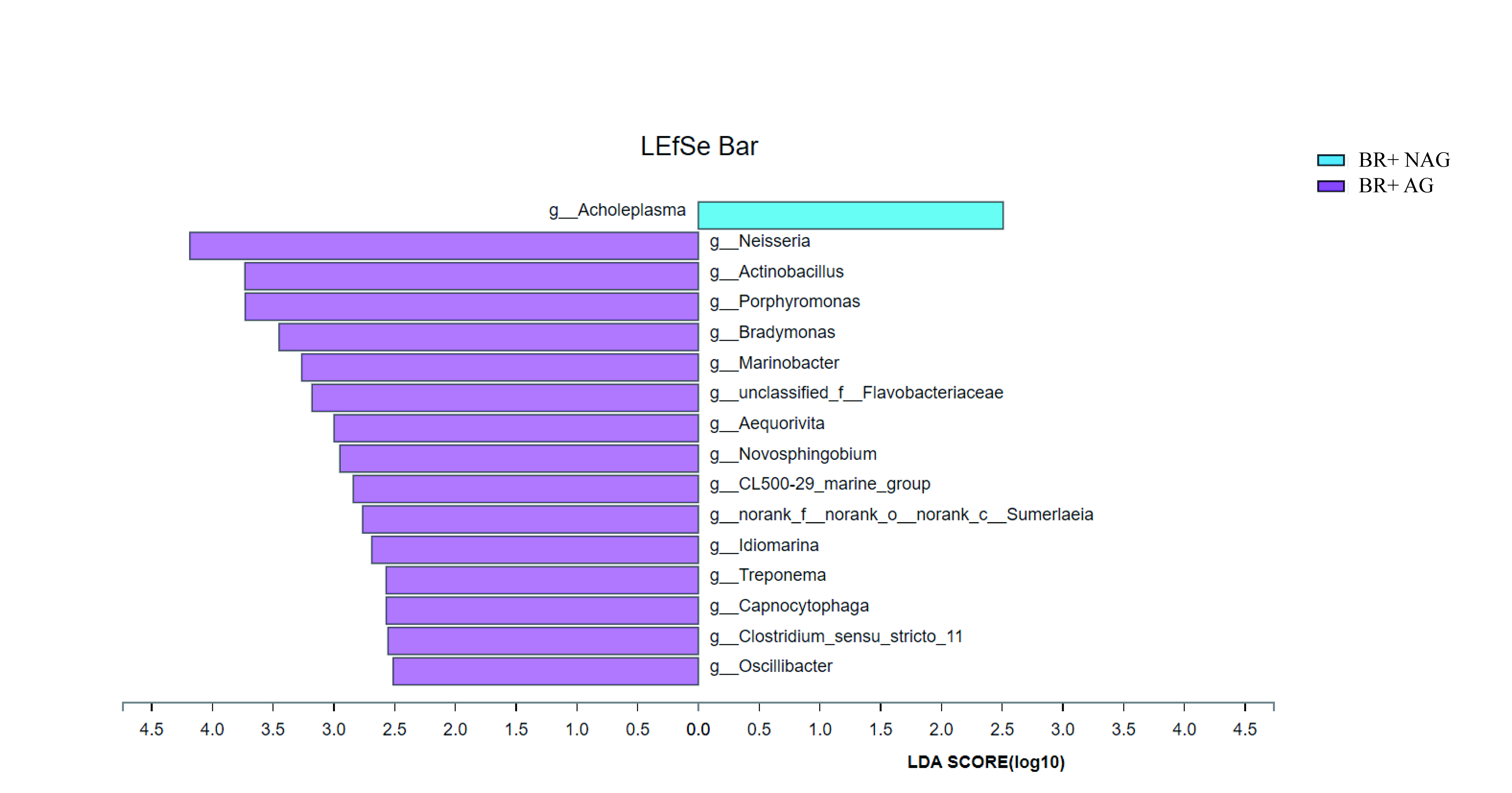

Supplement: Supplementary Figure 5 — The most differentially abundant taxa between the BR+ NAG and BR+ AG groups using LEfSe analysis. [file Image_5.tif]

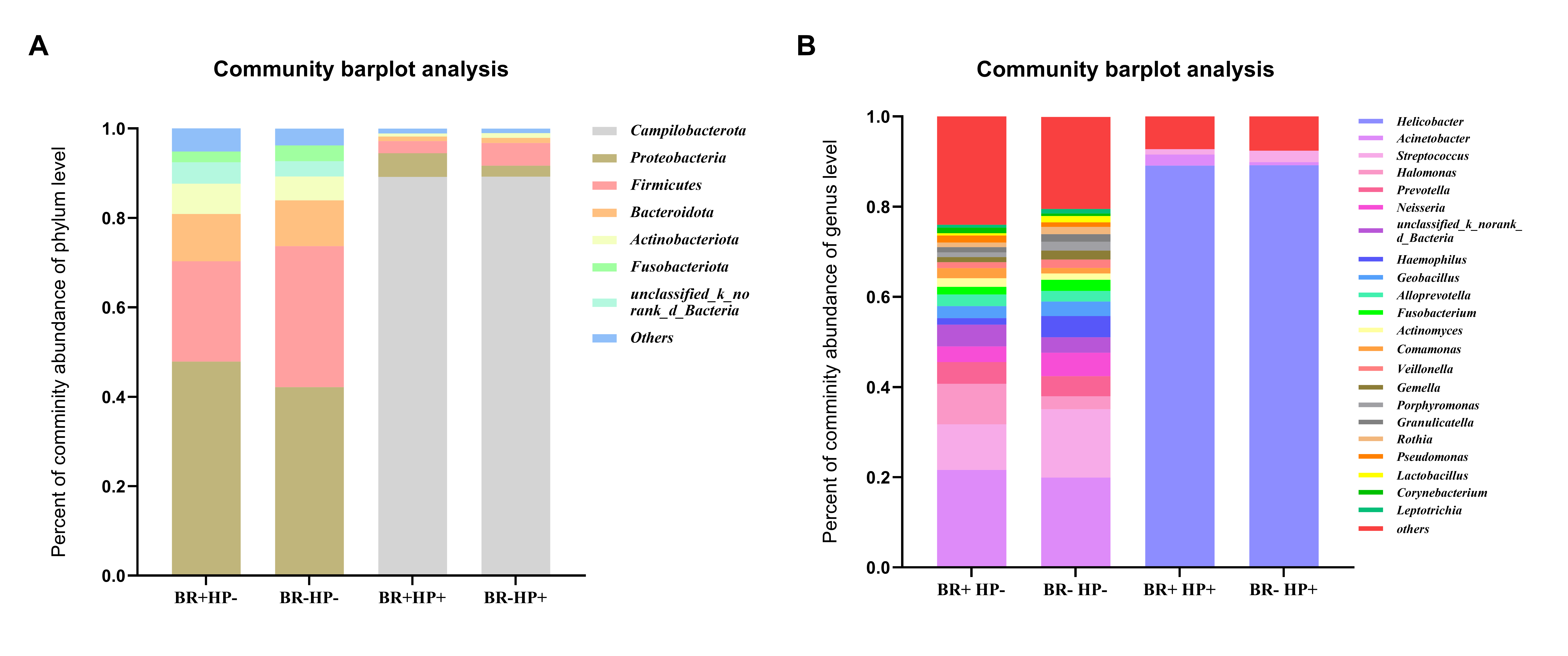

Supplement: Supplementary Figure 6 — The abundance of relative taxa in the gastric microbiota. Comparison of the abundance of relative taxa in the BR+ HP-, BR- HP-, BR+ HP+ and BR- HP+ groups at the (A) phylum and (B) genus level. [file Image_6.tif]
