# Supplementary material for: Bile reflux alters the profile of the gastric mucosa microbiota
Source: Front Cell Infect Microbiol. 2022 Sep 9;12:940687. doi: 10.3389/fcimb.2022.940687 (PMC9500345; doi:10.3389/fcimb.2022.940687)
Supplement: Supplementary file 7 [file Table_1.docx]

|  | **BR+ HP+** | **BR- HP+** | **BR+ HP-** | **BR- HP-** | ***P* value** |
| --- | --- | --- | --- | --- | --- |
|  | **(n=23)** | **(n=15)** | **(n=29)** | **(n=25)** |  |
| Age, y (mean±SD) | 48.95±14.41 | 42.07±17.01 | 47.90±13.74 | 50.84±12.70 | 0.301 |
| Sex, n (%) |  |  |  |  | 0.530 |
| Male | 15(65.2) | 8(53.3) | 15(51.7) | 11(44.0) |  |
| Female | 8(34.8) | 7(46.7) | 14(48.3) | 14(56.0) |  |

**Table S1 Population characteristics based on H. pylori infection and bile reflux grouping**

H pylori infection determined by 16 s RNA sequencing
