# Supplementary material for: Bile reflux alters the profile of the gastric mucosa microbiota
Source: Front Cell Infect Microbiol. 2022 Sep 9;12:940687. doi: 10.3389/fcimb.2022.940687 (PMC9500345; doi:10.3389/fcimb.2022.940687)
Supplement: Supplementary file 8 [file Table_2.docx]

|  | **BR+ NAG** | **BR- NAG** | **BR+ AG** | **BR- AG** | ***P* value** |
| --- | --- | --- | --- | --- | --- |
|  | **(n=17)** | **(n=14)** | **(n=13)** | **(n=11)** |  |
| Age, y (mean±SD) | 40.18±11.79 | 45.29±12.01 | 58.00±8.73 | 57.90±10.08 | <0.001^*φ^ |
| Sex, n (%) |  |  |  |  | 0.751 |
| Male | 8(47.06) | 5(35.71) | 7(53.85) | 6(54.55) |  |
| Female | 9(52.94) | 9(64.29) | 6(46.15) | 5(45.45) |  |

**Table S2 Population characteristics based on gastric atrophy and bile reflux grouping**

*BR+ NAG vs. BR- NAG *P*=0.243; ^φ^BR+ AG vs. BR- AG *P*=0.243
